# The effects of language proficiency and awareness of time limit in animated vs. text-based situational judgment tests

**DOI:** 10.1186/s12909-024-05513-z

**Published:** 2024-05-15

**Authors:** Mathis Rabe, Oana R. Gröne, Charlotte von Bernstorff, Mirjana Knorr

**Affiliations:** 1 Department of Biochemistry and Molecular Cell Biology, University Medical Center Hamburg-Eppendorf, N30, Martinistraße 52, Hamburg, 20246 Germany; 2 Department of Business Psychology, BSP Business and Law School, Calandrellistraße 1-9, Berlin, 12247 Germany

**Keywords:** Situational judgment test, Video-based, Animation, Text-based, Time limit, Language proficiency, Subgroup differences, Medical school admission

## Abstract

**Background:**

Situational Judgment Tests (SJTs) are commonly used in medical school admissions. However, it has been consistently found that native speakers tend to score higher on SJTs than non-native speakers, which can be particularly problematic in the admission context due to the potential risk of limited fairness. Besides type of SJT, awareness of time limit may play a role in subgroup differences in the context of cognitive load theory. This study examined the influence of SJT type and awareness of time limit against the background of language proficiency in a quasi high-stakes setting.

**Methods:**

Participants (*N* = 875), applicants and students in healthcare-related study programs, completed an online study that involved two SJTs: one with a text-based stimulus and response format (HAM-SJT) and another with a video-animated stimulus and media-supported response format (Social Shapes Test, SST). They were randomly assigned to a test condition in which they were either informed about a time limit or not. In a multilevel model analysis, we examined the main effects and interactions of the predictors (test type, language proficiency and awareness of time limit) on test performance (overall, response percentage).

**Results:**

There were significant main effects on overall test performance for language proficiency in favor of native speakers and for awareness of time limit in favor of being aware of the time limit. Furthermore, an interaction between language proficiency and test type was found, indicating that subgroup differences are smaller for the animated SJT than for the text-based SJT. No interaction effects on overall test performance were found that included awareness of time limit.

**Conclusion:**

A SJT with video-animated stimuli and a media-supported response format can reduce subgroup differences in overall test performance between native and non-native speakers in a quasi high-stakes setting. Awareness of time limit is equally important for high and low performance, regardless of language proficiency or test type.

**Supplementary Information:**

The online version contains supplementary material available at 10.1186/s12909-024-05513-z.

## Background

Situational Judgment Tests (SJTs) have long been a common tool [1] for assessing non-academic abilities [2], such as collaboration and communication [3] or professional behavior [4]. To accomplish this, candidates are provided with scenarios that represent interpersonal situations and are usually asked to evaluate several possible behavioral responses [2]. As personnel selection tools, SJTs have been used for a long time [5] and two decades ago, Lievens and Coetsier [6] proposed SJTs also as a tool for assessing applicants to medical schools. Internationally, SJTs are increasingly being used in the field of medicine and demonstrate predictive validity for non-academic abilities [2]. Examples of SJTs currently used for medical school admissions are the Casper [3] the SJT subtest of UCAT [4] and the German Hamburger Situational Judgment Test (HAM-SJT) [7].

A weakness of many SJTs is that they often tend to show subgroup differences in test performance. For example, many studies have found variations among subgroups based on gender in favor of women over men or based on race in favor of whites over Blacks, Hispanics and Asians [8–10]. However, in addition to these well-studied subgroups, some studies have also found differences between subgroups based on language factors. For instance, native speakers tend to score better on SJTs than non-native speakers [11–13].

The presence of subgroup differences in SJTs, particularly in the context of medical school admission, could be problematic for two reasons. First, it could raise concerns about test fairness. Although subgroup differences do not necessarily imply a limitation of fairness, they can be an indicator that test fairness might be threatened and warrant further investigation (e.g., analysis of differential item functioning or predictive fairness) [14, 15]. A fair test ensures consistent constructs and scores across all test takers in the intended population, without favoring or disadvantaging individuals based on irrelevant characteristics [16]. Subgroup differences could indicate that, aside from non-academic competencies, other skills such as language skills are unintentionally measured and that the construct that the test aims to assess is measured differently in each group. Thus, a threat to test fairness could also be a threat to test validity [16]. Second, subgroup differences could also counteract widening participation policies of an institution. Many medical schools aim to promote diversity among future medical students and medical professionals [17, 18]. Even if a test demonstrates promising evidence for its predictive validity, medical schools might still desist from using this test if applicants from minority groups significantly underperform and their chances of entering medical school are thus reduced (i.e., “diversity-validity dilemma”) [10, 19]. Therefore, a selection test should ideally be developed in way that it both demonstrates predictive validity and reduces subgroup differences in order to promote diversity.

### Predictor method factors of SJTs

In research, testing procedures such as SJTs are often considered holistically and their modularity is simplified [20]. A more differentiated view is provided by the “predictor method factors”, which allow to describe and distinguish between various concrete examples of test methods such as SJTs on the basis of their features. Following Lievens and Sackett [20], the components of a SJT can be broken down into the following seven factors: (1) stimulus format, (2) contextualization, (3) stimulus presentation consistency, (4) response format, (5) response evaluation consistency, (6) information source, and (7) instructions (For a more detailed description of the predictor method factors, see [20]).

In the following, we will focus on the stimulus and response format, since these method factors can provide an approach to reduce variations among subgroups [21–23]. According to Lievens and Sackett [20], the stimulus format describes the way in which the test stimuli (for SJTs usually a scenario of a social interaction) are presented to the participants. The response format describes how the participants are required to respond to the test stimuli. Both predictor method factors can be divided into different categories. The most common stimulus and response formats for SJTs are text-based, e.g., the UCAT SJT subtest [4] or the HAM-SJT [7]: A scenario is described in text form and so are response options, which are then assessed for appropriateness.

Apart from fully text-based SJTs, there are also a number of SJTs that cover other categories of stimulus and response formats, for example, stimulus formats that include videos with actors [11], animated characters [22], or geometric shapes [24]. There are also SJTs that use free text responses [10], text-based evaluations accompanied by audio [22], or single Choice text-reduced responses supported by symbols [24] to respond to stimuli. Casper [3] is a popular example of a SJT that uses a video stimulus format for most of the scenarios and a free text response format.

### SJT approaches to reducing subgroup differences based on language proficiency

Some studies have shown that using video-based stimulus formats instead of text-based ones can reduce subgroup differences for ethnicity and gender [21]. Additionally, using less text-based response formats, such as audiovisual constructed design, has also been found to be effective to reduce subgroup differences between minority and majority populations [23].

Initial studies also indicate that a reduction in text quantity in SJTs may lead to a reduction of subgroup differences based on language factors. For example, Karakolidis et al. [22] discovered somewhat smaller subgroup differences based on language proficiency in their SJT with video-animated stimuli and media-supported response format compared to a completely text-based SJT. However, these results were inconclusive in that language proficiency and reading comprehension correlated with test performance in both SJTs. Karakolidis et al. [22] explain the reduction of subgroup differences according to the theory of cognitive load [25]. Based on this reasoning, they assume that SJTs with video stimuli could reduce the excessive demands on the working memory through language-reading comprehension of the participants which could have a positive effect on the comprehension of the material [22]. Taking this assumption further, we could argue that non-native speakers might have parts of their cognitive capacity tied up by the translation process due to the limitations of working memory whereas native speakers can fully concentrate on the task. Such confounding effect of test scores based on language proficiency is a general weakness of text-heavy items and procedures that have already been found in other selection contexts and instruments [26, 27].

Brown et al. [28] also found a reduction of subgroup differences in their 2019 developed Social Shapes Test (SST) [24] compared to completely text-based SJT. The SST presents animated and text-free videos of simple geometric shapes without the use of verbal cues and a short question about them. The response format consists of concise simple statements about the content of a particular video and partly contains graphics of geometric shapes. The reduction of subgroup differences that Brown et al. [28] found for the SST were related to country of birth, in favor of people who were born in an English-speaking country compared to people who were not. However, they did not find differences between native and non-native English speakers in either the completely text-based SJT or the SST. Brown et al. [28] emphasized that the statistical power to detect the effects of these variables was limited due to the very small proportion of non-native English speakers (7,5%) in their sample.

### Awareness of time limit

Due to the selection context, SJTs are mostly conducted in a high-stakes setting. There is a lot of research comparing different aspects of high-stakes vs. low-stakes settings for SJTs (e.g., [29, 30]). However, when conducting SJTs in a high-stakes setting, there can be various factors in addition to the actual processing of the test material that place additional demands on cognitive resources, such as a time limit. Earlier studies indicated correlations between time pressure and test performance of students in other performance-based procedures [31]. In a high-stakes setting, the factor of time limit may have a special impact on non-native speakers. For example, when it comes to General Mental Ability tests, differences in test performance between native and non-native speakers have been found under time pressure [32]. Besides, one aspect that has been less explored is the awareness of time limit. Thus, the amount of information about the time limit could influence the level of stress and therefore the binding of cognitive resources. Following the cognitive load theory, the underlying process is the limitation of the cognitive resources available to a person at any given time. When cognitive load increases, the resources required to process a stimulus become more limited [33]. A constant reminder of the time limit could therefore intensify the awareness of time limit, thereby triggering more stress which in turn could bind more cognitive resources than a less intensive reminder of the time limit.

### Present study

The present study examined the influence of awareness of time limit and language proficiency in the HAM-SJT with text-based stimuli and response format (hereby referred to as text-based SJT) and the SST with video-animated stimulus and a media-supported response format (hereby referred to as animated SJT) in a quasi high-stakes setting. The aim was to examine the potential of animated SJTs using videos with animated geometric shapes to minimize subgroup differences based on language proficiency in the context of medical school admission in contrast to a completely text-based SJT. For transparency, our hypotheses and research questions were preregistered. All documents, including the preregistration, the data analysis R code and a template for data request can be retrieved from the Open Science Framework (OSF) project via https://osf.io/j87q5/.

In a first step, we aimed to (partially) replicate the findings of Brown et al. [28] on a larger sample with a higher proportion of non-native speakers in the German context of medical school admissions. Following the theory of cognitive load [25], processing the test material might require more cognitive effort for non-native speakers and thus engage more cognitive capacity that is no longer available for processing the test material. As a result, native speakers should perform better than non-native speakers. This is suggested by the findings of Graupe et al. [11] and Patterson et al. [12, 13] who found subgroup differences between native and non-native speakers in favor of native speakers in SJTs. In addition, this binding of cognitive resources should be more pronounced in text-based SJTs than in animated SJTs. This should reduce subgroup differences based on language proficiency in animated SJTs. The findings of Karakolidis et al. [22], who found smaller subgroup differences in their SJT with video-animated stimuli compared to a completely text-based SJT, also support this. This led to the following hypotheses:

- **Hypothesis 1a:** Regardless of test type (text-based vs. animated), native speakers will achieve higher test performance in the SJTs than non-native speakers.
- **Hypothesis 1b:** In the HAM-SJT (text-based), there will be larger subgroup differences in test performance between native and non-native speakers, in favor of native speakers, than in the SST (animated).

In the second step, we examined the influence of the awareness of time limit in the context of SJTs. In line with the cognitive load theory [25], the awareness of time limit could engage cognitive capacities that are no longer available to process the task and thus affect test performance.

This is supported, for example, by a study of Onwuegbuzie and Seaman [31], who found associations between time pressure and student test performance. In addition, this effect may be associated with an increased cognitive load on the text-based SJT for non-native speakers in addition to prolonged text processing. Here, the awareness of time limit may influence the relationship between language level and test performance. It may reinforce possible interaction effects and act as a moderator. In support of this assumption, Talento-Miller et al. [32] found that different language groups perform differently under a time limit in the context of General Mental Ability tests. These results might also be found in other performance-based procedures and thus be transferable to SJTs. Due to the lack of research on the awareness of time limit in general and especially in the context of SJT, this investigation primarily relies on theoretical considerations that suggest interactions but does not provide a definitive prediction of their direction. Therefore, instead of hypotheses, the following research questions were derived:

- **Research Question 1a:** What influence does awareness of time limit have on test performance?
- **Research Question 1b:** Does awareness of time limit moderate the relationship between language proficiency and test performance?
- **Research Question 1c:** Does awareness of time limit moderate the relationship between language proficiency and test performance depending on test type?

In the third step, we aimed to explore how awareness of time limit, test type and language proficiency affects response behavior. Response behavior was measured by the proportion of answered items and was considered as an additional operationalization of test performance, in addition to the overall test performance. The additional cognitive load [25] that non-native speakers might expand when processing text-heavy test materials compared to native speakers could lead to different response behaviors and thus influence the number of items processed. Therefore, native speakers might be able to process more items than non-native speakers. These subgroup differences may be stronger in the case of the text-based HAM-SJT than of the animated SST. For example, studies have found evidence that a time limit on a task can influence, among other things, risk decision-making processes in gambling (e.g., [34]) and response heuristics depending on whether the information is presented in textual or pictorial form [35]. Because of the proximity to the operationalization and because the hypothesis generation and the formulation of the research questions argumentatively follow those of the overall test performance, the hypotheses (H2a, H2b) and research questions (RQ2a, RQ2b, RQ2c) were derived and tested analogously to the analysis of the overall test performance.^1^

## Method

**Fig. 1:**
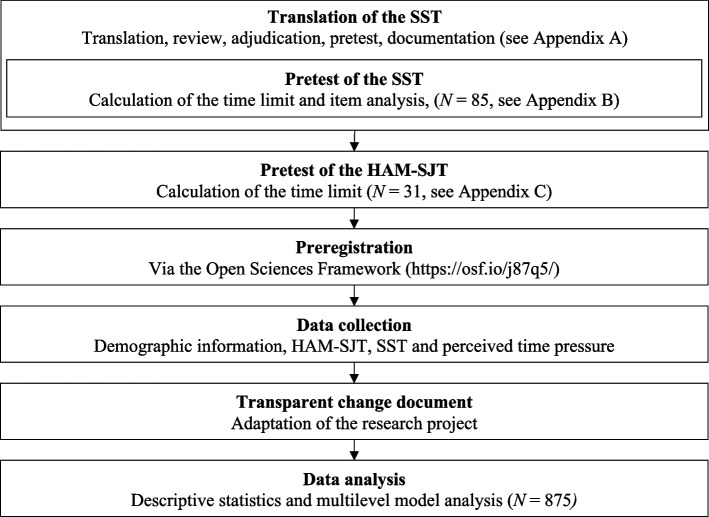
Flowchart of the study process

The present study was preceded by a number of steps, such as the preregistration of the study, the translation of the SST and two pretests. An overview of the entire study process can be seen in the flow chart in Fig. 1.

### Sample and procedure

Recruitment was done via a circular email among 19,799 participants of the research project of the German Student Selection Network (Studierendenauswahl-Verbund, stav). The database primarily includes applicants and students in healthcare-related study programs (e.g., medicine, dentistry, midwifery).

Participants completed the study via an online survey created with LimeSurvey (Version 5.5.0 [36]) using their own internet-enabled devices, accessed through a link in an email. They first filled out demographic information, completed the HAM-SJT and a German version of the SST (see Appendix A), then rated their perceived time pressure. The order of HAM-SJT and SST, the sequence of items within each test and the assignment to the awareness of time limit test condition or no awareness of time limit test condition were randomized. Initially, the test condition was designed to compare performance under time pressure versus a non-time pressure condition. A programming error resulted in the timer being hidden rather than turned off, which prevented participants from seeing it. However, the timer continued to run in the background and terminated the SJT without warning after a certain amount of time. This required an adaptation of the research design and question. The transparent change document on OSF provides further details regarding the adaptation of the research design and research questions.

In both SJTs, all items were presented on one page. Test instructions were shown on a separate page before the SJT as well as on top of the page with the actual test items (for the instruction we used for the SST, see [37]). In the SST, participants had the option to view the videos as many times as they liked. The participants were not able to switch back and forth between the SJTs.

To create a quasi high-stakes situation, different aspects were considered: processing time was tightly limited and incentive systems were used to motivate the participants to perform well.

The time limit occurred at the test level and was determined by pretesting based on the 25% percentile of processing time. Participants had 750 seconds for the SST (see Appendix B) and 960 seconds for the HAM-SJT (see Appendix C). In contrast to the no awareness of time limit condition, the awareness of time limit condition contained a visible timer between all items and at the beginning and end of the page.

As an incentive system, participants received a report on their performance within their test condition group. In addition, vouchers for a wide range of online stores were awarded based on performance. In total, 200 € was awarded twice, 100 € four times, 50 € ten times and 25 € 60 times.

### Measures

#### SST

The SST consists of video-animated stimuli, including 23 items as well as 4 attention check items, and a media-supported, single-choice response format with 23 items as well as 4 attention check items [24]. Each item consists of a short 10 to 23 seconds video, a question about what is happening in the video and four response options. Each video simulates a social interaction between colored geometric shapes (yellow plus, orange x, purple star, red square and blue triangle). The videos show a range of behaviors, such as bullying, comforting or playing [28]. Examples of items can be found in Brown et al. [28] and are freely available at OSF (https://osf.io/sqxy6). The correct responses were determined by the test authors [24]. Unanswered questions were given a score of zero in the SST.

In previous studies, the SST had a moderate internal consistency of $\alpha = 0.67$ [24] to $\alpha = 0.71$ [28] and an item difficulty of *p* = .70 (range: 0.35 to 0.96) [24]. We translated the SST into German (see Appendix A) following the TRAPD approach (see [38, 39]). The German SST showed an internal consistency of $\alpha = 0.59$ and the item difficulty of *p* = .72 (range: 0.33 to 0.99) in a pretest (see Appendix B).

#### HAM-SJT

**Fig. 2:**
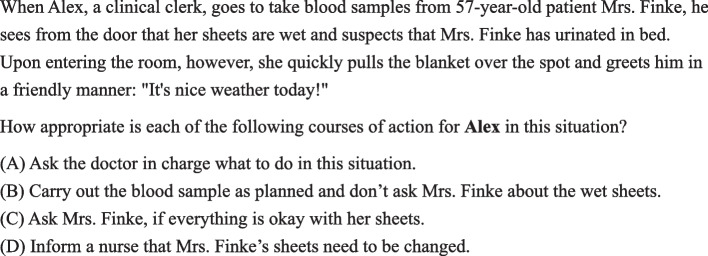
Example of HAM-SJT scenario with associated items

The HAM-SJT, piloted in 2016 [7] and introduced into high-stakes selection in 2020, has a text-based stimulus and response format. It is currently the only SJT used for high-stakes selection of medical students in Germany [7]. All items are written from a third-person perspective. Scenarios are set in a medical context but can be answered without prior knowledge (see Fig. 2 for an example scenario with associated items). The test version for this study contained 18 situations and 75 behavioral responses (three to five per situation). The participants were asked to rate the appropriateness of each response for the situation on a 4-point scale from 1 (very appropriate) to 4 (very inappropriate). Expert ratings serve as comparative values. To account for individual response styles, raw item values are intra-individually standardized [40]. The sum of the squared differences of all behavioral responses to expert values is calculated and linearly transformed to associate higher scores with better performance [41]. Unanswered items were assigned the raw score with the highest distance to the expert score which also had a negative impact on the final score. In previous studies, the HAM-SJT had a moderate internal consistency of $\alpha =0.67$ [41] to $\alpha = 0.70$ [42].

#### Subjectively perceived time pressure

The subjective time pressure was assessed individually for the HAM-SJT and the SST using two items specifically developed for this purpose. For both tests the item was phrased as following: “In the [test type], I was under a lot of time pressure to complete all the questions”. The answer was given on a 5-point Likert scale from 1 (completely disagree) to 5 (completely agree).

#### Demographics

The following demographic characteristics were collected: gender, age, citizenship, birth in a German-speaking country, mother tongue, second mother tongue for German native speakers, German language skills for non-German native speakers, type and average grade of higher education entrance qualification, current occupation as well as study program and number of semesters for students.

### Analysis

Based on the results of Brown et al. [28], an a priori power analysis for a multilevel modeling analysis ($f^{2}$ = 0.02, *df* = 6, $\alpha = 0.05$, $\beta = 0.80$, icc = 0.05) was conducted using the package sjstats [43] in R (Version 4.3.0 [44]). This resulted in a required minimum sample size of *N* = 688 participants.

Data analyses were performed using R (Version 4.3.0 [44]) with the packages lme4 [45] and lmerTest [46]. The significance level was set at *p* < .05. A multilevel analysis was used to test the hypotheses and examine our research questions. Two models were calculated. The first model was calculated for overall test performance scores as outcome variable. For this purpose, SST and HAM-SJT test scores were standardized across individuals. The second model was calculated for the response percentage as outcome variable, using the proportion of items completed per test type. A random effect was set for participants to control for the fact that participants performed both SJTs. To check the legitimacy of the random factor, the intraclass correlation coefficients (ICC) were calculated. As recommended by LeBreton and Senter [47] we used 0.05 as benchmark for the ICC value above which we deemed the use of a multilevel model with a random factor as justified.

Means, standard deviations and a Pearson correlation matrix were calculated for all relevant predictors as well as internal consistency (Cronbach’s alpha) for the HAM-SJT (based on the deviation value of each item) and the SST.

Main effects were tested for language proficiency (H1a) and awareness of time limit (RQ1a). Interaction effects were tested between language proficiency and test type (H1b), between language proficiency and awareness of time limit (RQ1b) as well as between language proficiency, awareness of time limit and test type (RQ1c). Additionally, the main effect for the test type was also assessed to check for interacting effects.

Because multiple studies have shown that women tend to perform better in SJTs than men (e.g., [48]), we controlled for gender. Based on the results of Brown et al. [28], who found subgroup differences in favor of people born in an English-speaking country, we controlled for people born in a German-speaking country. Since the perception of time pressure is essential in the context of our study and we wanted to know whether awareness of time limit had an impact beyond perceived time pressure, we controlled for this.

## Results

### Sample description

A total of 1084 people completed the full survey. We excluded 227 participants from the analysis because they answered more than one attention check item incorrectly on the SST or completed the SST faster than possible (470s were needed to watch all videos). The participants were also excluded if they exceeded the maximum completion time on one of the SJTs, reported technical problems or did not complete the survey. In case of multiple participation, only the first participation was included in the analysis. Participants who stated their gender as “diverse” were also excluded from the analysis, as the small number ($n = 7$) would have led to model bias. This resulted in a final sample size of *N* = 857 participants.

The participants were mostly female (*n* = 610, 71.12%) and born in a German-speaking country (*n* = 794, 92.35%) with a mean age of 22.64 years (range: 16 to 47, *SD* = 3.75). Students accounted for 65.81% of the sample with an average of 4.1 semesters (range: 1 to 17, *SD* = 2.19). Of these, the majority were medical students (67.91%) or dental students (6.92%).

The native language of most participants was German (*n* = 760, 88.68%), of which 90 participants (11,84%) reported a second native language. Most German native speakers were female ($n = 540, 71.05\%$). Of the 97 participants (11.32%) who were not native German speakers, 73 (75.26%) estimated their German language skills as C2 (based on the Common European Framework of Reference for Languages), 22 (22.68%) as C1, one (1.03%) as B2 and one (1.03%) as A1. Most of the non-native speakers were also female (n = 70, 72.16%). Compared to the results of Groene et al. [49] about the demographics of medical students in Germany, our sample has a similar proportion of females (71.1%) compared to the medical students in Germany (73.3%). In addition, our sample has a slightly higher proportion of non-native German speakers (11.32%) compared to medical students in Germany (6.2%) [49] but appears to be lower if compared to potential future applicants, i.e. current secondary school students in the city where this study was conducted (20.5% to 40.3% depending on school type) [50]. This indicates that although the ratio of native to non-native speakers in the current study is representative of the current population of medical students in Germany, it does not reflect the population of future potential applicants. Table 1 summarizes the sample description across the awareness of time limit test condition.

**Table 1:** Sample description across the awareness of time limit test conditions

	Awareness of time limit		No awareness of time limit		Total sample	
	*n*	%	*n*	%	*n*	%
Gender						
Female	325	70.96	285	72.52	610	71.18
Male	139	29.04	108	27.48	274	28.82
Born in a German-speaking country						
Yes	431	92.88	363	92.37	794	92.35
No	33	7.11	30	7.63	63	7.35
German as native language						
Yes	417	89.87	343	87.28	760	88.68
No	47	10.13	50	12.72	97	11.32
Total sample	464	53.03	396	45.26	875	100.00

### Descriptive statistics

The internal consistency of the SST was $\alpha = 0.64$ and participants achieved a mean score of *M* = 15.57 (range: 4 to 23, *SD* = 3.45) out of a possible maximum score of 23. The internal consistency of the HAM-SJT was $\alpha = 0.92$ and participants achieved a mean score of *M* = 2.43 (range: 0 to 2.95, *SD* = 0.51). Correlations between all study variables included in the main analysis are reported in Table 2.

**Table 2:** Descriptive statistics and correlation matrix

Variable	*M* ^3^	*SD*	$\varvec{\alpha }$	1	2	3	4	5	6	7	8
1. SST	15.57	3.45	0.64	–							
2. HAM-SJT^1^	2.43	0.51	0.92	$$0.26^{***}$$	–						
3. Female gender (vs. Male)	0.71	0.45		$$-0.03$$	$$0.12^{***}$$	–					
4. Born in a German-speaking country	0.93	0.26		$$0.10^{**}$$	$$0.13^{***}$$	$$-0.04$$	–				
5. Language proficiency	0.89	0.32		$$0.11^{**}$$	$$0.21^{***}$$	$$-0.01$$	$$0.55^{***}$$	–			
6. Awareness of time limit	0.54	0.50		$$0.28^{***}$$	$$0.26^{***}$$	$$-0.03$$	0.01	0.04	–		
7. Perceived time pressure in SST^2^	3.14	1.39		$$-0.28^{***}$$	$$-0.04$$	$$-0.02$$	0.00	0.02	$$0.14^{***}$$	–	
8. Perceived time pressure in HAM-SJT^2^	2.95	1.47		$$-0.05$$	$$-0.41^{***}$$	$$-0.14^{***}$$	$$-0.06$$	$$-0.08^{*}$$	$$0.13^{***}$$	$$0.21^{***}$$	–

*N* = 857; *r* = Pearson correlation coefficients; * *p* < .05, ** *p* < .01, *** *p* < .001; Language proficiency (0 = non-native speaker, 1 = native speaker); Awareness of time limit (0 = no awareness, 1 = awareness)

^1^Inverted score, divided by the number of items

^2^Scale for perceived time pressure: "In the [test type], I was under a lot of time pressure to complete all the questions"

^3^For the categorical variables (3,4,6), mean values are replaced by proportion values

### ICC

The calculation of the ICC resulted in a value of ICC = 0.264 using overall test performance as the outcome (ICC = 0.370 using response percentage as the outcome). The values show that 26.4% of the variance of overall test performance (37% for response percentage) is explained by the random factor and thus justifies the use of a multilevel model.

### Effects on overall test performance

All tested main and interaction effects for overall test performance are reported in Table 3. In line with hypothesis 1a, multilevel analysis revealed a significant main effect for language proficiency on overall test performance ($b = 0.59, p < .001$). This indicates that native speakers in general achieve better overall test performance than non-native speakers.

**Table 3:** Estimation of the fixed effects for the overall test performance

Effect	*b*	SE	*t*	*df*	*p*
Intercept	-0.25	0.14	-1.79	1,307.67	.074
Female gender (vs. Male)	0.06	0.05	1.14	854.38	.254
Born in German speaking country	0.15	0.10	1.47	855.74	.143
Time pressure	-0.26	0.01	-17.71	1,703.50	< .001
Language proficiency	0.59	0.13	4.64	1,370.94	< .001
Awareness of time limit	0.82	0.14	6.00	863.59	< .001
Test type	0.23	0.11	2.04	855.43	.041
Language proficiency $\times$ test type	-0.26	0.13	-2.04	857.35	.042
Language proficiency $\times$ awareness of time limit	-0.25	0.15	-1.69	990.10	.091
Language proficiency $\times$ awareness of time limit $\times$ test type	0.10	0.08	1.24	855.01	.216

*N* = 857; Language proficiency (0 = non-native speaker, 1 = native speaker); Awareness of time limit (0 = no awareness, 1 = awareness); Test type (0 = HAM-SJT, 1 = SST)

**Fig. 3:**
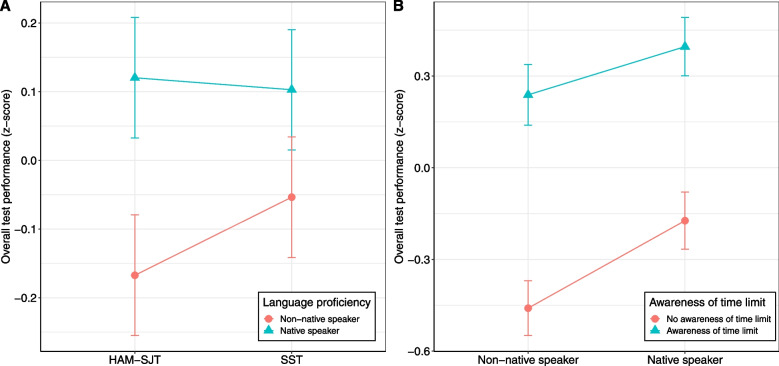
Interaction plot between language proficiency and test type (**A**), as well as awareness of time limit for overall test performance (**B**); error bars show a 95% confidence interval

Supporting hypothesis 1b, the interaction between language proficiency and test type was also significant ($b = -0.26, p = .042$). This indicates that the differences in overall test performance between native and non-native speakers were greater in the HAM-SJT than in the SST (see Fig. 3A).

In response to research question 1a, awareness of time limit was found to be significantly associated with overall test performance ($b = 0.82, p < .001$). This indicates that participants who were aware of the time limit had higher test scores (see Fig. 3B).

Neither the two-way interaction (RQ1b) between language proficiency and awareness of time limit ($b = -0.25, p = .091$; see Fig. 3B), nor the three-way interaction (RQ1c) between language proficiency, awareness of time limit and test type were significant ($b = 0.10, p = .216$). Thus, no difference in overall test performance was found between native and non-native speakers as a function of awareness of time limit or awareness of time limit and test type.

### Effects on response percentage

All exploratively tested main and interaction effects for response percentage are reported in Table 4. The main effects for (H2a) language proficiency ($b = 0.11, p < .001$) and (RQ2a) awareness of time limit ($b = 0.16, p < .001$) became significant. This indicates that native speakers and participants who were aware of the time limit answered proportionally more items than non-native speakers and participants who were not aware of the time limit. The interaction (H2b) between language proficiency and test type ($b = - 0.06, p < .001$) became significant as well as the interaction (RQ2b) between language proficiency and awareness of time limit ($b = - 0.07, p < .001$). For a visualization see Appendix D, Fig. D1. This indicates that the SST compared to the HAM-SJT, as well as awareness of time limit compared to no awareness of time limit, resulted in smaller subgroup differences. The three-way interaction (RQ2c) between language proficiency, awareness of time limit and test type was also significant ($b = 0.06, p < .001$). This indicates that the three predictors, interacting with each other, influence the proportion of items answered. Thus, the interaction between language proficiency and awareness of a time limit differs depending on the test type. For a visualization see Appendix D, Fig. D2.

**Table 4:** Estimation of the fixed effects for the response percentage

Effect	*b*	SE	*t*	*df*	*p*
Intercept	0.87	0.02	40.78	1,256.54	< .001
Female gender (vs. Male)	0.01	0.01	0.80	854.58	.426
Born in German speaking country	0.01	0.02	0.81	858.24	.416
Time pressure	-0.05	0.00	-20.50	1,689.85	< .001
Language proficiency	0.11	0.02	5.71	1,286.77	< .001
Awareness of time limit	0.16	0.02	7.59	868.24	< .001
test type	-0.01	0.02	-0.34	855.53	.736
Language proficiency $\times$ test type	-0.06	0.02	-3.17	857.44	.002
Language proficiency $\times$ awareness of time limit	-0.07	0.02	-2.78	968.24	.006
Language proficiency $\times$ awareness of time limit $\times$ test type	0.06	0.01	4.98	855.12	< .001

*N* = 857; Language proficiency (0 = non-native speaker, 1 = native speaker); Awareness of time limit (0 = no awareness, 1 = awareness); Test type (0 = HAM-SJT, 1 = SST)

## Discussion

The present study was designed to examine the influence of the awareness of time limit and language proficiency in the text-based HAM-SJT and the video-based SST in a quasi high-stakes setting. There are four main findings from our research. First, there are subgroup differences in SJTs between native and non-native speakers for the overall test performance. Second, the use of animated rather than text-based SJTs can reduce these subgroup differences. Third, awareness of time limit is important for the overall test performance, but not in interaction with the language proficiency or the test type. Fourth, the response behavior is influenced by language proficiency, the awareness of time limit and the test type.

### Overall test performance

**Fig. 4:**
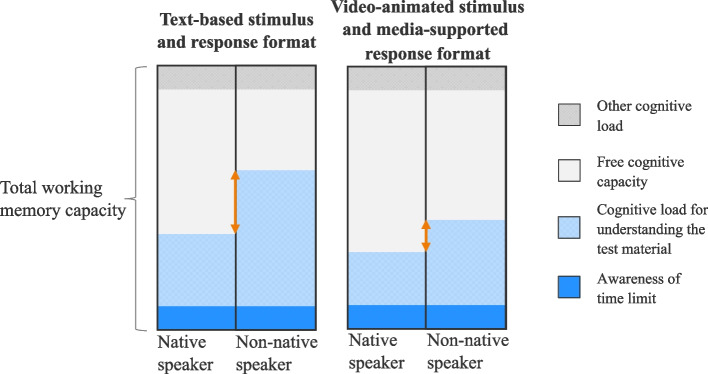
Visualization of the theory of congitive loading against the background of different stimulus and response formats, adapted from Moreno and Park [33]

In our study, we found subgroup differences based on language proficiency in overall test performance on the SJT in favor of native versus non-native speakers (H1a). This finding is consistent with the current state of research [11–13]. Furthermore, our study expands on a previously understudied research field about the influence of stimulus and response format on language proficiency-related subgroup differences in overall test performance in SJTs. Our findings that video-animated stimuli can reduce subgroup differences (H1b) confirm and expand findings by Brown et al. [28] who found that the SST could reduce subgroup differences for people born in an English-speaking country, but not for non-native English speakers. The larger proportion of non-native speakers in our study compared to Brown et al. [28] may explain why we were able to find an effect for language proficiency. Our results are also in line with research by Karakolidis et al. [22] and support the idea to relate these subgroup differences to cognitive load theory. Accordingly, changing the stimulus and response format may reduce cognitive load for non-native speakers in language-reading comprehension. This could potentially reduce subgroup differences. The reason for this reduction is likely to be that non-native speakers benefit more from such a change than native speakers, who have to expend fewer cognitive resources to complete the test. Figure 4, visualizes this theory of cognitive load applied to hypotheses H1a and H1b in the context of text-based stimulus and response formats compared to video-animated stimuli and media-supported response formats.

Our study also shows that awareness of time limit is positively related to overall test performance (RQ1a). According to the theory of cognitive load [25], the awareness of a time limit could trigger stress, which in turn could tie up cognitive capacities that are no longer available for processing the task. Our findings suggest that other factors arising from the awareness of time limit might have a greater influence than the bound resources. One possible factor could be processing strategies such as time management. Time management, made possible by the awareness of the exact time limit, could outweigh the influence of the induced stress by time limit on the overall test performance. In general, the results highlight the importance of being aware of time limit. In doing so, they emphasize the common practice in selection procedures, where participants are precisely informed about the available time.

The research questions (RQ1b, RQ1c) aimed to explore whether awareness of time limit serves as a means to diminish subgroup differences. Additionally, the investigation sought to determine the varying impact based on test type or whether, for instance, non-native speakers are more influenced by this awareness compared to native speakers. We could not find any interactions between awareness of time limit and language proficiency or language proficiency and test type (RQ1b, RQ1c). Thus, we could not confirm the assumption of a moderating effect of awareness of time limit in dependence on language proficiency. Awareness of time limit seems to influence overall test performance for native and non-native speakers alike. This indicates that the effects found by Talento-Miller et al. [32] that different language groups perform differently under a time limit in the context of General Mental Ability tests are not fully transferable to SJTs, even though this is also a proficiency test. The underlying process between awareness of time limit and time pressure may therefore be different than suspected. Rather than tying up cognitive resources, awareness of time constraints seems to promote better performance. The awareness of a time limit thus does not seem to have a potentiating or stronger effect for non-native speakers and help both native and non-native speakers alike to perform better, e.g., by giving them both the opportunity to make better use of time management.

### Response percentage

The explorative analysis of the influences on the response percentage suggests a great importance of language proficiency, awareness of time limit and test type. Both language proficiency (H2a) and awareness of time limit (RQ2a) are related to the response percentage. In addition, these factors, together with the test type, also interact with each other to affect the proportion of responses (H2b, RQ2b, RQ2c). With regard to language proficiency, we assume that these interactions can be explained by the theory of cognitive load in which non-native speakers have less resources available for responding and therefore answer fewer items. With regard to awareness of time limit, this underlines previous findings that have shown that time limits can influence response behavior in different ways (e.g., [34, 35]).

Of particular interest are the results for research questions RQ2b and RQ2c, which examine the interaction with awareness of time limit and its dependence on test type. In contrast to the results for overall test performance, the percentage of items answered appears to depend on both awareness of a time limit and test type, and to differ between native and non-native speakers. Thus, the differences in the percentage of items answered between native and non-native speakers depend on whether they were aware of the time limit or not. Moreover, this difference is greater in the HAM-SJT than in the SST. This could be explained by the fact that non-native speakers benefit more than native speakers when they are aware of a time limit and can adapt their response strategies accordingly in order to answer more items. For instance, non-native speakers may be more likely to take risks and use exclusion or guessing strategies when aware of time constraints [34]. Although native speakers may be able to cope better with the material due to the lower cognitive load of the tasks, they may use such strategies less than non-native speakers, which means that non-native speakers benefit more from the awareness of a time limit. This is emphasized, among others, by the fact that this effect is greater in the HAM-SJT than in the SST, which could be due to the text-heavy format of the stimuli and response formats in the HAM-SJT, which non-native speakers find more difficult to deal with.

The analysis of response percentage is more inductive and exploratory, albeit hypothesis-driven. Therefore, the results should be interpreted with caution. However, the results show the extent to which response percentage, and perhaps response behavior in general, can be influenced.

### Limitations and future research

First, in the current study, awareness of time limit was assessed only dichotomously (awareness vs. no awareness). This limits the interpretability of the results regarding the relevance of awareness of time limit to the extent that it is only possible to analyse whether awareness of a time limit has an impact, but not which degree of awareness of time limit would be optimal. In practice, however, the more interesting question, which can be built on the findings here, is the extent to which information about the time limit should be provided. Future studies should explore awareness of time limit in a hierarchical manner and investigate how different degrees of clarification of time limit (e.g., variation in the number and size of timers and variation in the number and clarity of statements in the instruction that indicate a time limit) could impact overall test performance and subgroup differences.

Second, in the no awareness of time limit test condition, participants who did not manage to complete the first SJT within the time limit were warned of a possible time limit by the sudden termination of the SJT after a certain time. It is possible that this warning affected the processing of the second SJT. Therefore, it might be that the participants worked on the second test under stress, as they expected a similar termination as in the first test. However, due to the randomized order of the SJTs, this effect should be negligible. In addition, there is the possibility that some participants knew each other and talked about the study. These participants might have thus been warned beforehand about the time limit. However, given the participation rate (1084 out of 19,799 individuals) as well as the random assignment to different time limit conditions this probably only affected a negligible number of participants.

Third, although we used a performance-based incentive system, our results might differ to an actual high-stakes selection setting. Our study shows an almost identical mean test score ($M = 15.57, SD = 3.45$) as the study by Brown et al. [28] ($M = 15.39, SD = 3.49$) despite a different test language, sample and a low-stakes setting. This could be explained by a high robustness of the procedure to external influences but could also mean, that our study setting was similarly perceived as low stakes.

Fourth, future hypothesis-driven research should examine of whether response strategies differ systematically between native and non-native speakers, and whether this reflects cultural difference or language proficiency. Additionally, it’s important to analyze the influence of time limit on whether the awareness of a time limit or the time limit itself moderates this effect.

Fifth, it should be noted that the pretest sample only consisted of psychology students, on whose basis the time limits were calculated. This could be problematic as psychology students may perform differently in SJTs than other health-care students. For instance, they may be faster at answering questionnaires due to their frequent exposure to them as part of their studies.

Sixth, it should be investigated in future studies whether the results found are actually due to cognitive differences. This could be done by using control variables. For example, by measuring general cognitive ability using the grades of the university entrance qualification, or by using a short intelligence test to measure fluid intelligence, which according to Wilhelm et al. [51] is strongly related to working memory capacity.

Seventh, it should be noted that the German version of the SST has only been pretested in a small sample of psychology students and its validity and retest reliability have not yet been evaluated. Further studies are necessary to confirm its psychometric properties in larger and more diverse samples and to test its relationship to conceptually related or different constructs.

Finally, the highly significant, albeit rather weak, correlation (*r* = 0.26, *p* = < 0.001) between the SST and the HAM-SJT (see Table 2) indicates that the SST measures similar aspects to the HAM-SJT. However, test developers of the SST could already show promising convergent validity with some SJTs and other performance measures of social competence [28, 37]. For its use in the medical school admission context, however, more studies are required first. For example, performance at Objective Structured Clinical Examinations (OSCE), which also assess communication skills [52], could be used as outcome criteria to examine predictive validity. A closer examination of face validity is crucial, as some of the qualitative comments in our study suggest that participants were unable to understand the meaning of the test and preferred a contextualized test such as the HAM-SJT. But, it is important to consider familiarity with the test material. For instance, many participants were likely familiar with the HAM-SJT, while the SST was likely unknown to most, if not all, of them.

### Practical implications

SJTs with video-animated stimuli and media-supported response format such as the SST could help reduce subgroup differences between native and non-native speakers in medical school admission. Furthermore, they can be a cost-effective alternative to video-based procedures with human actors (e.g., [53]) and at the same time prevent biases due to the appearance of the actors [28].

The results also underline the importance of the awareness of time limit for all participants, without native or non-native speakers particularly benefiting from it. It thus supports the common practice in selection procedures in which participants are precisely informed about the time available. However, this also raises the question of the appropriate time limit for conducting SJTs. An implementation without time pressure could perhaps reduce the subgroup differences between native speakers and non-native speakers even further, as the factor of the processing speed of the test material would no longer apply in this case. This could be realized, for example, by a significantly longer processing time.

In our study, the SST tended to be completed better than the HAM-SJT. This is indicated by the main effect of test type in favor of the SST for overall test performance (see Table 3). As a result, there is a risk that the SST will not differentiate well in the upper range of performance. In the context of medical school admission, where such differentiation is desirable, items with lower difficulty should be developed before the test is used as a selection tool.

The results can be an important source of information for admissions committees. For example, although SJTs are already increasingly used in practice [1], the question is to what extent there is awareness and ideas about how they can be used. The results presented here can provide support in both cases. First, they illustrate the subgroup differences that can arise, for example, on the basis of language proficiency, which can lead to discrimination against non-native speakers, and can therefore raise awareness of this issue. Second, they offer ways of dealing with subgroup differences, especially for admissions committees that develop SJTs themselves, as the results suggest that the use of animation-based procedures can help to reduce subgroup differences.

## Conclusion

The present study provides an initial evaluation of an animated SJT using simple geometrical shapes (SST) in a quasi high-stakes setting. The SST can reduce subgroup differences in overall test performance between native and non-native speakers and thus if used in selection potentially increase cultural diversity based on native language in healthcare programs in line with higher education policy interests. Awareness of time limit is equally important for good performance, regardless of language proficiency or test type, and should be well communicated according to common practice.

### Supplementary Information

Supplementary Material 1.

## Data Availability

Data are available via kontakt@projekt-stav.de upon reasonable request and with permission of the stav. Additional material including a preregistration, R code, a data request form and the online supplement can be retrieved from the Open Science Framework via https://osf.io/j87q5/.

## Abbreviations

HAM-SJT: Hamburger Situational Judgment Test
ICC: intraclass correlation coefficient
OSF: Open Science Framework
SJT: Situational Judgment Test
SST: Social Shapes Test

## Footnotes

1
**H2a:** Regardless of test type (text-based vs. animated), native speakers will achieve higher response percentage in the SJTs than non-native speakers. **H2b:** In the HAM-SJT (text-based), there will be larger subgroup differences in response percentage between native and non-native speakers, in favor of native speakers, than in the SST (animated). **RQ2a:** What influence does awareness of time limit have on response percentage? **RQ2b:** Does awareness of time limit moderate the relationship between language proficiency and response percentage? **RQ2c:** Does awareness of time limit moderate the relationship between language proficiency and response percentage depending on the test type?
