# Supplementary material for: The effects of language proficiency and awareness of time limit in animated vs. text-based situational judgment tests
Source: BMC Med Educ. 2024 May 15;24:540. doi: 10.1186/s12909-024-05513-z (PMC11097494; doi:10.1186/s12909-024-05513-z)
Supplement: Supplementary file 1 — Supplementary Material 1. [file 12909_2024_5513_MOESM1_ESM.pdf]

Appendix A German translation process of the SST

Prior to the actual study, it was necessary to adapt the original English-language SST to the German language. For this purpose, the TRAPD approach [see 1, 2] was used. TRAPD is an acronym that describes the stages of the process: translation, review, adjudication, pretesting and documentation.

Translation. A student of business psychology (M.Sc.) (a native German speaker with good English skills) and a linguist in English and French, as well as a communication scientist and an expert for underrepresented populations in medical studies (good German and English skills), independently produced parallel translations between February 27 and March 12, 2023.

Review. The review process on March 13, 2023, involved the translators and a reviewer who is a native speaker of the target language with good English skills, and possesses knowledge of survey research methodology. They discussed the advantages and disadvantages of each item translation and agreed on the most appropriate versions.

Adjudication. The adjudicator, a trained and experienced expert in aptitude assessment, evaluated the final versions against the original items, considering the original translations, the final version, and any comments made during the process.

Pretesting. The translated SST was tested via an online survey on a sample of psychology students to assess the extent to which the translations were understood. The participants had a chance to win one of 8 vouchers which were raffled among those who have fully participated in the pretest. Participants who had an account to the research participation and management tool "SONA systems" received an allowance in the form of 0.5 credits. The data collection took place between March 24 and April

6, 2023 (two weeks). The psychometrics of the translation were examined quantitatively using Cronbach's alpha, item discriminant power, item difficulty and response frequencies. Qualitative feedback on the translation was obtained, in which the participants were asked to assess the general comprehensibility of the questionnaire and to give any suggestions for improvement.

Documentation. The process and its results were properly documented (https://osf.io/j87q5/). The Excel file documents the parallel translations, substantial comments during the review, the results of the review, the reviewer's adjustments and the final version of the German translation.

Appendix B Pretest results of the German translation of the SST

After the completion of the data collection (see Appendix A), the data of 135 participants were available. The data of the participants who did not complete the test (n = 20), who answered more than one attention check item incorrectly (n = 10), who did not study psychology (n = 1) or whose processing time was shorter than the total time needed to watch all videos (470s) were excluded from the evaluation (n = 11). This left a sample of N = 100 participants for the analysis. For more details of the sample, see Table B1.

Table B1 Descriptive statistics for sample

Baseline characteristic	Participants	
	n	%
Type	Psychology students	
Gender		
Female	84	84.00
Male	16	16.00

Note. N = 100. Participants were between 18 and 57 years old (M = 23.60, SD = 4.42).

The N = 100 participants scored between 8 and 22 ($M = 16.51, SD = 3.02$) out of a possible 23 points. The summed item completion time ranged from 531.49s to 5141.42s ($M = 1131.57, SD = 733.84$). Excluding $n = 15$ outliers (identified as values outside 1.5 times the interquartile range from the quartiles), $n = 85$ participants had a mean processing time of M = 889.95s (SD = 212.04) and a 25% percentile of 750.63s or 12.5 min. A summary of the quantitative results of the pretest is presented in Table B2.

The qualitative question at the end of the study, regarding the evaluation of the translation, was answered by n = 14 participants. The translation was considered adequate by n = 6 respondents, n = 1 respondent described the task as "sometimes

unclear what to pay attention to", $n = 2$ would have been pleased with the ability to switch between items and $n = 1$ pointed out that the comma placement was sometimes not quite correct but without affecting comprehension. The remaining $n = 4$ respondents did not make any statements about the appropriateness of the translation but rather included general expressions of interest in the procedure.

Looking at the results, it is noticeable that the German adaptation of the SST has a lower internal consistency ($\alpha = 0.59$) than in previous studies of the English version ($\alpha = 0.67$ [3], $\alpha = 0.71$ [4]). The reasons for this may be due to differences in the sample or the circumstances of the testing or may even indicate that the translation was not satisfactory. However, the lower value in general may be due to the presumed multidimensionality of the SST [3]. Future studies should also look at test-retest reliability as suggested by Corstjens et al. [5] for multidimensional SJTs. The negative, albeit weak, discriminatory power of the SST12 of r_{it} = -0.03 should also be noted. This can also be explained by multidimensionality. Nevertheless, the composition of the items should be examined more closely and critically reflected upon in future studies. The test difficulty is similar to the original study by Brown et al. [3] with $p = 0.70$ (range: 0.35-0.96), although it should be noted that some of the items have a high test difficulty (SST04, SST06, SST11, SST23) and are therefore probably too easy, at least for the selection context. In order to be able to better refer to the results from the previous studies, it was decided to use the SST in its entirety in the further course of this study.

Table B2 Quantitative summary of the pretest for the German translation of the SST

Item name	Video file	α	α, if omitted	r_{it}	p	Correct	Response frequency			
							A	B	C	D
SST02	SS1		0.59	0.09	0.41	C	34	18	41	7
SST03	Sick		0.58	0.17	0.45	D	24	22	9	45
SST04	Exclusion		0.57	0.25	0.95	A	95	0	2	3
SST05	Matchmaking		0.56	0.29	0.80	B	1	80	12	7
SST06	Bullying 1		0.58	0.25	0.99	B	0	99	1	0
SST07	Comfort		0.58	0.11	0.78	C	0	12	78	10
SST08	Friendship		0.57	0.23	0.79	D	17	1	3	79
SST09	Mocking		0.58	0.18	0.55	C	11	26	55	8
SST10	Baby		0.56	0.30	0.74	C	12	6	74	8
SST11	Line Cutting		0.57	0.24	0.94	A	94	0	1	5
SST12	Distraction		0.61	-0.03	0.61	B	1	61	19	19
SST13	Tricking		0.58	0.14	0.73	D	25	1	1	73
SST14	Couple		0.57	0.23	0.91	A	91	2	7	0
SST15	Juggling		0.56	0.31	0.79	D	13	6	2	79
SST16	Stealing		0.60	0.03	0.59	B	4	59	8	29
SST17	Duck Duck Goose		0.56	0.27	0.65	A	65	18	9	8
SST18	Sad		0.56	0.27	0.84	D	4	2	10	84
SST19	FB		0.55	0.37	0.77	D	11	7	5	77
SST20	Scaring		0.59	0.08	0.33	A	33	12	30	25
SST21	Strangers		0.59	0.12	0.55	D	7	34	4	55
SST22	Cyberball		0.58	0.15	0.76	A	76	23	0	1
SST23	Helping		0.57	0.23	0.93	A	93	0	4	3
SST24	SS2		0.55	0.33	0.61	D	3	37	2	61
Total		0.59			0.72					

Note. $N = 100$; r_{it} = coefficient of discriminatory power; p = item difficulty

Appendix C Pretest results of the HAM-SJT

The data collection took place between April 25 and April 26, 2023. After the completion of the data collection, the data of $N = 32$ participants were available. For more details, see Table C3. The summed processing time of the items of the $N = 32$ participants ranged from 712.82 to 1590.75s ($M = 1069.89, SD = 190.83$). Excluding the outliers ($n = 1$), the mean processing time of $n = 31$ participants was $M = 1053.09$s ($SD = 168.21$) and a 25% percentile of 968.81s or approximately 16 min.

Table C3 Descriptive statistics for sample

Baseline characteristic	Participants	
	n	%
Type	Psychology students	
Gender		
Female	26	83.87
Male	5	16.13

Note. $N = 31$. Participants were between 21 and 28 years old ($M = 24.36, SD = 1.78$).

Appendix D Visualization for effects on response percentage

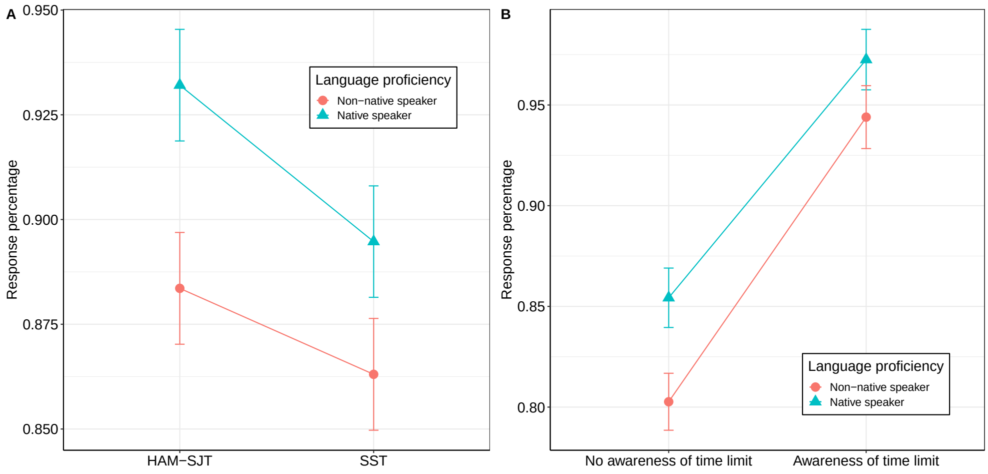

Fig. D1 Interaction plot between language proficiency and test type (A), as well as awareness of time limit (B) for response percentage; error bars show a 95% confidence interval.

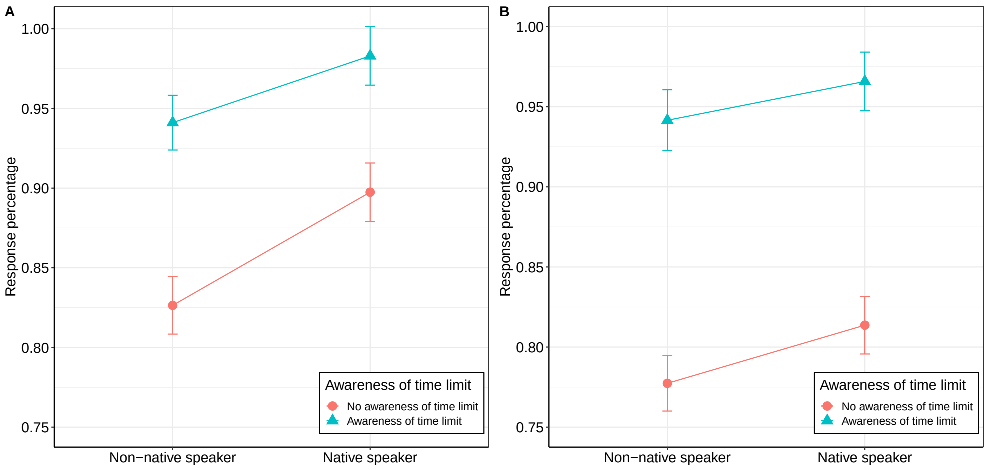

Fig. D2 Interaction plot between awareness of time limit for response percentage for the HAM-SJT (A) and the SST (B); error bars show a 95% confidence interval.

References

[1] European Social Survey. ESS Round 10 translation guidelines; 2020.

[2] Harkness JA. Questionnaire Translation. In: Harkness JA, Van de Vijver FJR, Mohler PP, editors. Cross-cultural Survey Methods. New York: Wiley; 2003. p. 35–56.

[3] Brown MI, Ratajska A, Hughes SL, Fishman JB, Huerta E, Chabris CF. The social shapes test: A new measure of social intelligence, mentalizing, and theory of mind. Personality and Individual Differences. 2019;143:107–117. https://doi.org/10.1016/j.paid.2019.01.035.

[4] Brown MI, Speer AB, Tenbrink AP, Chabris CF. Using game-like animations of geometric shapes to simulate social interactions: An evaluation of group score differences. International Journal of Selection and Assessment. 2022;30(1):167–181. https://doi.org/10.1111/ijsa.12375.

[5] Corstjens J, Lievens F, Krumm S. Situational judgement tests for selection. In: Goldstein HW, Pulakos ED, Passmore J, Semedo C, editors. The Wiley Blackwell Handbook of the Psychology of Recruitment, Selection and Employee Retention. 1st ed. Wiley; 2017. p. 226–246. Available from: https://onlinelibrary.wiley.com/doi/10.1002/9781118972472.ch11.
